# Functional Adrenocortical Adenoma in a Child with Beckwith–Wiedemann Syndrome

**DOI:** 10.1155/2021/5570267

**Published:** 2021-08-06

**Authors:** Leen Jamel Doya, Naya Talal Hassan, Hanin Ahmed Mansour, Mohammad Ahmad Almahmod Alkhalil, Abdul Alrahman Almahmod Alkhalil, Nada Mansour, Alaa Abdallah

**Affiliations:** ^1^Department of Pediatrics, Tishreen University Hospital, Lattakia, Syria; ^2^Department of Dermatology, Tishreen University, Latakia, Syria; ^3^Department of Pediatric Surgery, Tishreen University Hospital, Lattakia, Syria; ^4^Al-Hawash Private University, Homs, Syria

## Abstract

Beckwith–Wiedemann syndrome (BWS) is a rare congenital condition characterized by complex overgrowth of different body parts. Children with Beckwith–Wiedemann syndrome, particularly those with hemihypertrophy, experience an increased risk of developing benign and malignant tumors. This case report presents an 18-month-old girl with features suggestive of Beckwith–Wiedemann syndrome who developed pubic hair, high levels of testosterone, and DHEAS with normal cortisol and progesterone levels. Computed tomography revealed a left adrenal mass. Histopathological examination of the resected mass showed an adrenocortical tumor. Her postoperative evaluation showed normal testosterone and DHEAS levels. Early diagnosis and detection of intra-abdominal neoplasms in infants with Beckwith–Wiedemann syndrome are essential to avoid serious clinical complications.

## 1. Background

Beckwith–Wiedemann syndrome (BWS) is also termed EMG (exomphalos, macroglossia, and gigantism) syndrome. Historically, the first clinical description was in 1960 by John Bruce Beckwith and Hans-Rudolf Wiedemann [1]. The estimated population incidence of BWS is 1 in 13,700. Most of them (less than 85%) are sporadic, while less than 15% are familial with an autosomal dominant mode of inheritance, or chromosomal abnormalities (1%) on chromosome 11p15 [2].

BWS has a highly variable manifestation; abnormal growth is the most common feature which can result in macrosomia, hemihypertrophy, or enlarged tongue. Other presentations include abdominal wall defects, visceromegaly, ear lobe creases, cleft palate, renal alterations, refractory hyperinsulinemia, and polydactyly. Neurologic complications are rare [3]. BWS patients are at increased risk of developing both malignant and benign neoplasms, especially during the first eight years of life in cases of hemihypertrophy [4]. The overall tumor risk is about 5–10%; Wilm's tumor is the most common (52%) followed by hepatoblastoma (14%), neuroblastoma (10%), rhabdomyosarcoma (5%), and adrenal cortical carcinoma (3%) [2]. For this reason, screening for embryonic tumors is necessary for patients with BWS. Here, we report a case of a functional adrenocortical adenoma in an 18-month-old female with features suggestive of BWS, to emphasize the importance of detection of intra-abdominal neoplasms in BWS patients.

## 2. Case Presentation

An 18-month-old female child was referred with a 5-month history of excessive pubic hair (pubarche). She was born at full term by normal spontaneous vaginal delivery, following an uncomplicated pregnancy with no polyhydramnios. No congenital malformations were noticed at birth like exomphalos and macroglossia. She was noted to have left-sided hemihypertrophy involving the trunk and limbs without any management. She had neither hypoglycemic attacks nor omphalocele. Her birth weight was 4000 g (50^th^ percentile). There was no family history of genetic disorders.

On examination, her body weight was 15 kg (50^th^ percentile), the length 89 cm (50th percentile), temperature 37°C, oxygen saturation 98%, and arterial blood pressure 90/55 mm Hg. She looked well with whole left-sided hemihypertrophy, umbilical hernia, ear lobe creases, and pubic hair. She had no axillary hair growth, acne, breast enlargement, or vaginal bleeding. No rapid weight or height gain was noticed. Her Tanner staging was A1, P2, and B1. Laboratory investigation reports were as in Table 1.

**Figure 1:**
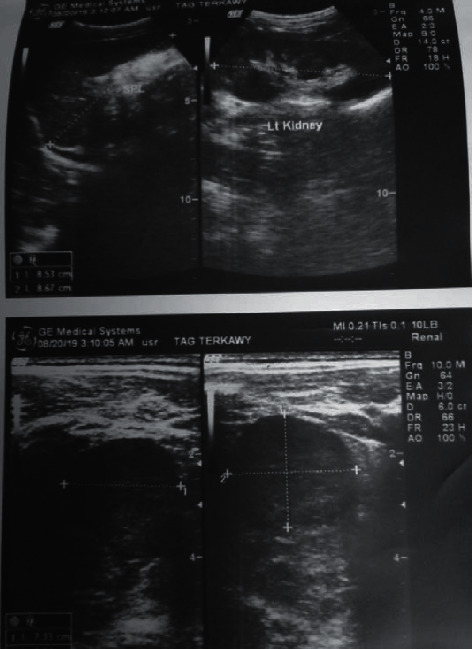
An abdominal ultrasound showed a well-defined rounded hypoechoic lesion with multiple tiny calcifications in the left suprarenal region measured 37 × 27 × 25 mm.

Echocardiography was normal. Left wrist X-ray showed a bone age of 3 years. The abdominal ultrasound showed a well-defined rounded hypoechoic lesion with multiple tiny calcifications in the left suprarenal region measured 37 x 27 x 25 mm **(**Figure 1). Computed tomography (CT) showed a 49 x 29 x 26 mm well-circumscribed solid mass in the left suprarenal region. Tumor marker, neuron-specific enolase (NSE), in blood and vanillylmandelic acid (VMA) in 24-hour urine were normal. Based on the above data, the interpretation was adrenal androgen-secreting tumor either adenoma or carcinoma.

**Figure 2:**
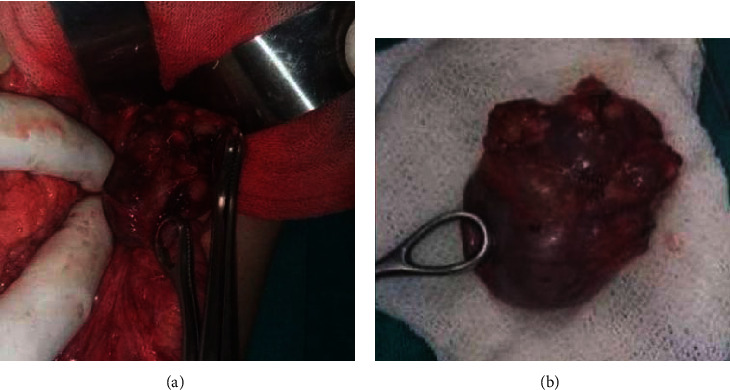
Grossly, encapsulated mass, no invasion to surroundings, size 5 × 4 × 2.8 cm.

Therefore, the patient underwent right open adrenalectomy: grossly encapsulated mass of size 5 x 4 x 2.8 cm and weighing 23.5 grams, with an outer glistening nodular surface, no invasion to surroundings (Figure 2). Histopathology revealed focally high-grade atypical (nuclear grades III and IV), mitotic activity >5/50 HPF, rare atypical mitosis, diffuse growth pattern, clear cells <25% of tumor size, and focal invasion of the adrenal gland capsule. Besides, there was hemorrhage and rare dystrophic calcification (Wieneke index criteria: 2/9) (Table 2). The tumor cells were negative chromogranin and sporadic positive of S100. Based on the weight, size of the tumor, microscopic features, and Wieneke criteria, a diagnosis of adrenocortical adenoma (ACA) was made. The patient was discharged 5 days after surgery, with no postoperative complications. The postoperative testosterone, DHEAS, 17-OH progesterone, cortisol, and LDH levels were found to be normal. We followed up the patient for 2 years postoperative. The patient is healthy, with a normal growth rate, and her blood measures remain normal until now (Table 3).

## 3. Discussion and Conclusion

The major and minor findings associated with BWS are important for the clinical diagnosis in the unavailability of genetic tests. A special clinical criterion of BWS containing cardinal and suggestive features that take points has been set. If the total points are greater or equal to 4 in the absence or negative of the genetic tests, the diagnosis of BWS is made [5] (Table 4). Due to the high risk of neoplasia in BWS, all of BWS's children should undergo cancer screening (alpha-fetoprotein (AFP) measurement every 2-3 months in the first 4 years of life for early detection of hepatoblastoma). Besides, an abdominal ultrasound is recommended every 3 months until at least eight years of age [1]. Our patient had features suggestive of BWS (left-sided hemihypertrophy, umbilical hernia, and ear lobe creases). No molecular test has been done in our case, so a clinical diagnosis has been proposed based on the presence of three criteria.

Adrenocortical tumors (ACT) are rare in children (less than 0.2% of all childhood tumors), and their incidence is increased by 40-fold in BWS patients. More than 90% of them are associated with the active secretion of adrenocortical hormones including androgens, estrogens, cortisol, and aldosterone. Most of these tumors have been suggested as carcinomas, and pure virilizing adenomas are rare [6]. In the literature review, there were about 4 cases that described the association between adrenocortical adenoma in BWS patients and most of them are older than 2 years [7–10] (Table 5).

Our case was not diagnosed as a case of BWS till 18 months of age and regular screening was not carried out. The child presented with only excessive pubic hair (pubarche), and this was the chief complaint that leads to the diagnosis of BWS.

Histopathologically, the tumor has a different pattern of growth that makes distinguishing between benign and malignant neoplasms difficult. Wieneke index, which requires histopathological findings, is employed to distinguish benign from malignant tumors. This classification is the most accurate score in children. Weineke score consists of the tumour size weight, size, tumor extension (invasion into the periadrenal soft tissue or adjacent organs, vena cava, venous, capsular), mitotic index, confluent necrosis, and atypical mitosis. The tumor is rendered as benign when the score is two or less [3-1].

In our case, considering the weight and size of the tumor, coupled with the microscopy, elevated hormonal levels, Wieneke score, and the clinical symptoms, a diagnosis of ACA was made. The tumor was completely removed without cortisol replacement.

A functional adrenocortical tumor has rarely been documented in a child, but it is increased in the presence of BWS. Early abdominal ultrasound is recommended in infants with BWS due to high suspicion for neoplasms. Early diagnosis and treatment are essential to avoid serious clinical complications.

## Figures and Tables

**Table 1:** The laboratory data of the case.

Test	Result	Normal range
WBC (10^3^/*μ*l)	9.3	(6.2–17)
Neutrophils (%)	70	(40–60)
Lymphocyte (%)	20	(20–40)
Hb (g/dl)	8.7	(11–13)
MCV (fl)	60	(70–85)
PLT (10^3^/*μ*l)	522	(150–450)
Urea (mg/dl)	30	(15–36)
Creatinine (mmol/L)	0.5	(0.5–1.3)
ESR (1 h, 2 h)	(18, 30)	(1–10)
CRP (mg/dl)	3	<5
Uric acid (mg/dL)	2.8	(2–5.5)
ALT (U/L)	20	(7–55)
AST (U/L)	15	(5–40)
Na (mmol/L)	135	(135–145)
K (mmol/L)	4.5	(3–4.5)
Urinalysis, culture, and sensitivity	Normal	—
Ferritin (ng/ml)	24	(15–400)
LDH (IU/L)	1032↑	(164–286)
17-OH progesterone (nmol/L)	4↔	(12–18)
Testosterone (ng/ml)	412↑	≤32.0
DHEAS (*μ*g/dl)	593↑	≤30.0
Cortisol at 6.0 am (*μ*g/dl)	10	4.0–20.0

WBC: white blood cell; HB: hemoglobin; MCV: mean corpuscular volume; RDW: red cell distribution width; PLT: platelets; ESR: erythrocyte sedimentation rate; CRP: C-reactive protein; LDH: lactate dehydrogenase; ALT: alanine aminotransferase; AST: aspartate aminotransferase; Na: sodium; K: potassium; DHEA: dehydroepiandrosterone.

**Table 2:** Wieneke index criteria in our patient.

Wieneke criteria	Our case
Tumor weight >400 g	23.5 g
Tumor size >10.5 cm	(5 × 4 × 2.8) cm
Extension into periadrenal soft tissues and/or adjacent organs	Not present
Invasion into vena cava	Not present
Venous invasion	Not present
Capsular invasion	Focal invasion
Presence of tumor necrosis	Not present
>15 mitoses per 20 HPF	>5/50 HPF
Presence of atypical mitotic figures	Rare

HPF: high-power fields.

**Table 3:** Preoperative and postoperative (after 5 days) hormonal profile.

Serum hormone	Preoperative level	Postoperative level	Reference range
LDH (IU/L)	1032↑	180	(164–286)
17-OH progesterone (nmol/L)	4↔	5	(12–18)
Testosterone (ng/ml)	412↑	20	≤32.0
DHEAS (*μ*g/dl)	593↑	28	≤30.0
Cortisol at 6.0 am (*μ*g/dl)	10	10	4.0–20.0

**Table 4:** BWS clinical diagnosis criteria.

Cardinal features (2) points	Suggestive features (1) points
Macroglossia Omphalocele	Large birth weight Facial nevus simplex
Lateralized overgrowth Hyperinsulinism	Polyhydramnios and/or placentomegaly
Multifocal/bilateral Wilms tumor or nephroblastomatosis pathogenic findings	Ear creases/pits Umbilical hernia and/or diastasis recti
	Organomegaly
	Embryonal tumors
	Transient hyperglycemia

**Table 5:** Cases of adrenocortical adenoma in BWS in the literature review.

Sbragia-Neto L et al. (2000), Brazil	2-year-old boy with BWS	Signs of virilization such as rapid growth and phallic enlargement with prepubertal testicular size	Elevated levels of androstenedione, dehydroepiandrosterone sulphate, and 17-ketosteroids with normal levels of cortisol	A right virilizing adrenal adenoma

Beauloye V et al. (2000), France	16-month-old girl with BWS	—	Elevated levels of 17-ketosteroids and androgens	Bilateral adrenal adenoma

Mizota M, et al. (2005), Japan	13-year-old girl with BWS	Clinical manifestation of virilization such as acne, voice change, clitoris hypertrophy, and overgrowth	High serum levels of DHEAS and testosterone	A right virilizing adrenal adenoma at age 6 and left adrenocortical adenoma at age 11

Elnaw E. A, et al. (2019), Sudan	4-year-old female with features suggestive of BWS	History of virilization	High levels of testosterone and DHEAS with normal cortisol level	Right adrenocortical adenoma

## Data Availability

All data generated or analyzed during this study are included in this article.
